# Supplementary figures and images for: Emergence of directional bias in tau deposition from axonal transport dynamics
Source: PLoS Comput Biol. 2021 Jul 27;17(7):e1009258. doi: 10.1371/journal.pcbi.1009258 (PMC8345857; doi:10.1371/journal.pcbi.1009258)

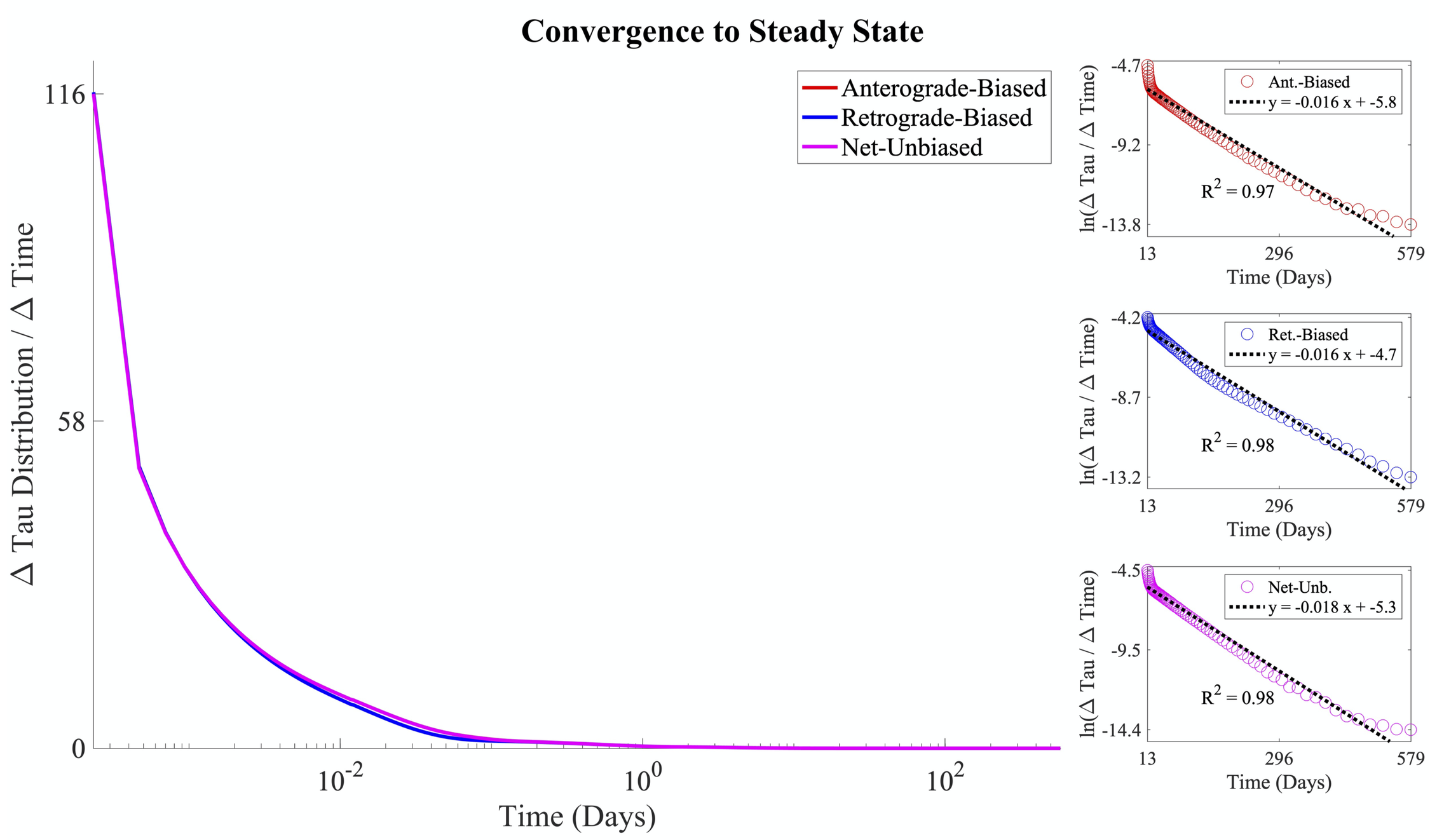

Supplement: S1 Fig — The rate of change of tau distributions (Eq 14) for the three parameter regimes explored in the main text (S1 Table) approaches zero within the time scale of the simulation. Notably, at mid-to-late model times, convergence is well approximated by an exponential, with similar rates of decay for all three parameter regimes (right panels). (TIF) [file pcbi.1009258.s003.tif]

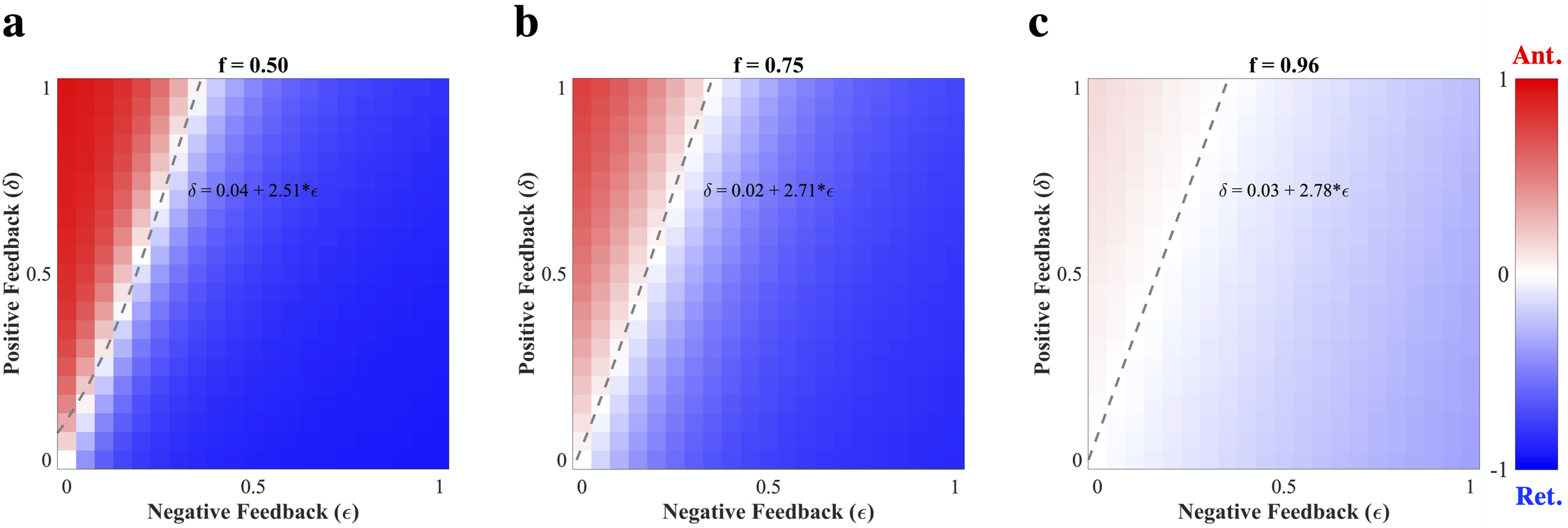

Supplement: S2 Fig — (a) Steady-state bias (postsynaptic SD tau—presynaptic SD tau / total SD tau; see Eq 15) across a range of δ and ϵ parameter values where all other parameter values are identical to those of the previous simulations. There is a zero-bias linear manifold that emerges, whose best-fit line has a slope of approximately 2.8. (b) Steady-state bias for the same range of δ and ϵ parameter values where aggregation rate (β) is doubled. The linear zero-bias manifold has a slope of ∼1.4, roughly half that of the original fragmentation rate. (c) Steady-state bias for the same range of δ and ϵ parameter values where β is halved. Here the slope of the linear manifold is ∼5.8, or approximately twice that of the original fragmentation rate. (TIF) [file pcbi.1009258.s004.tif]

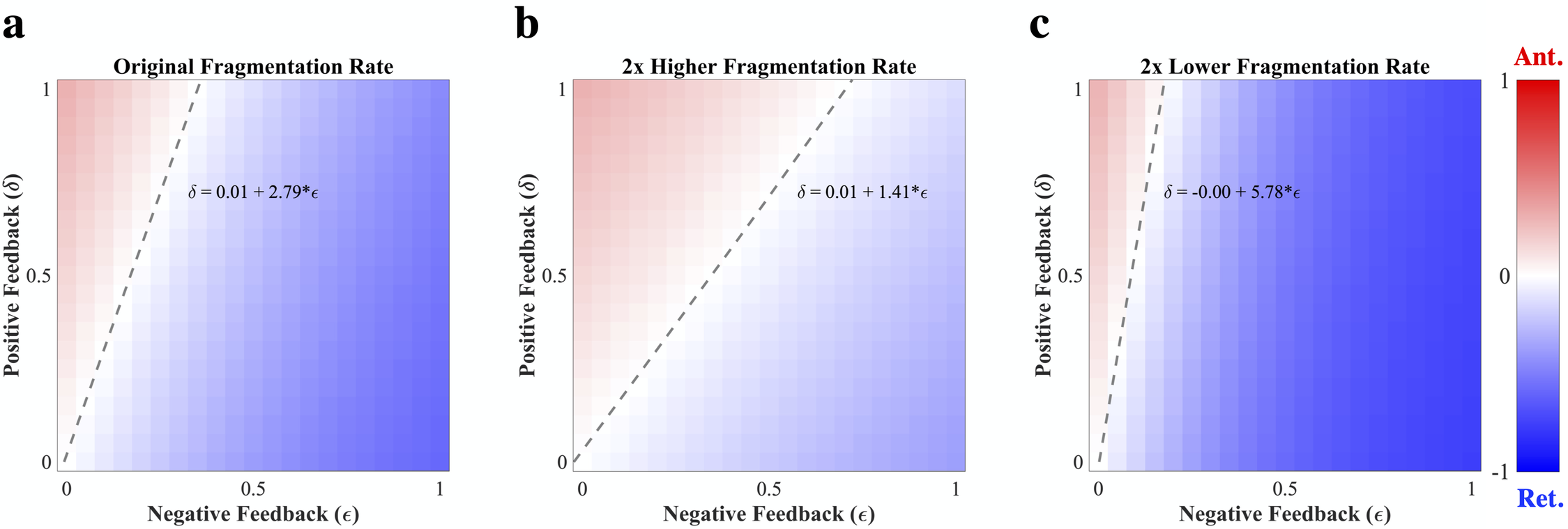

Supplement: S3 Fig — The overall strength of the net bias at any given pair of δ and ϵ values is inversely proportional to f, but it does not affect the line of zero bias (dashed lines), in contrast to γ and β. (TIF) [file pcbi.1009258.s005.tif]

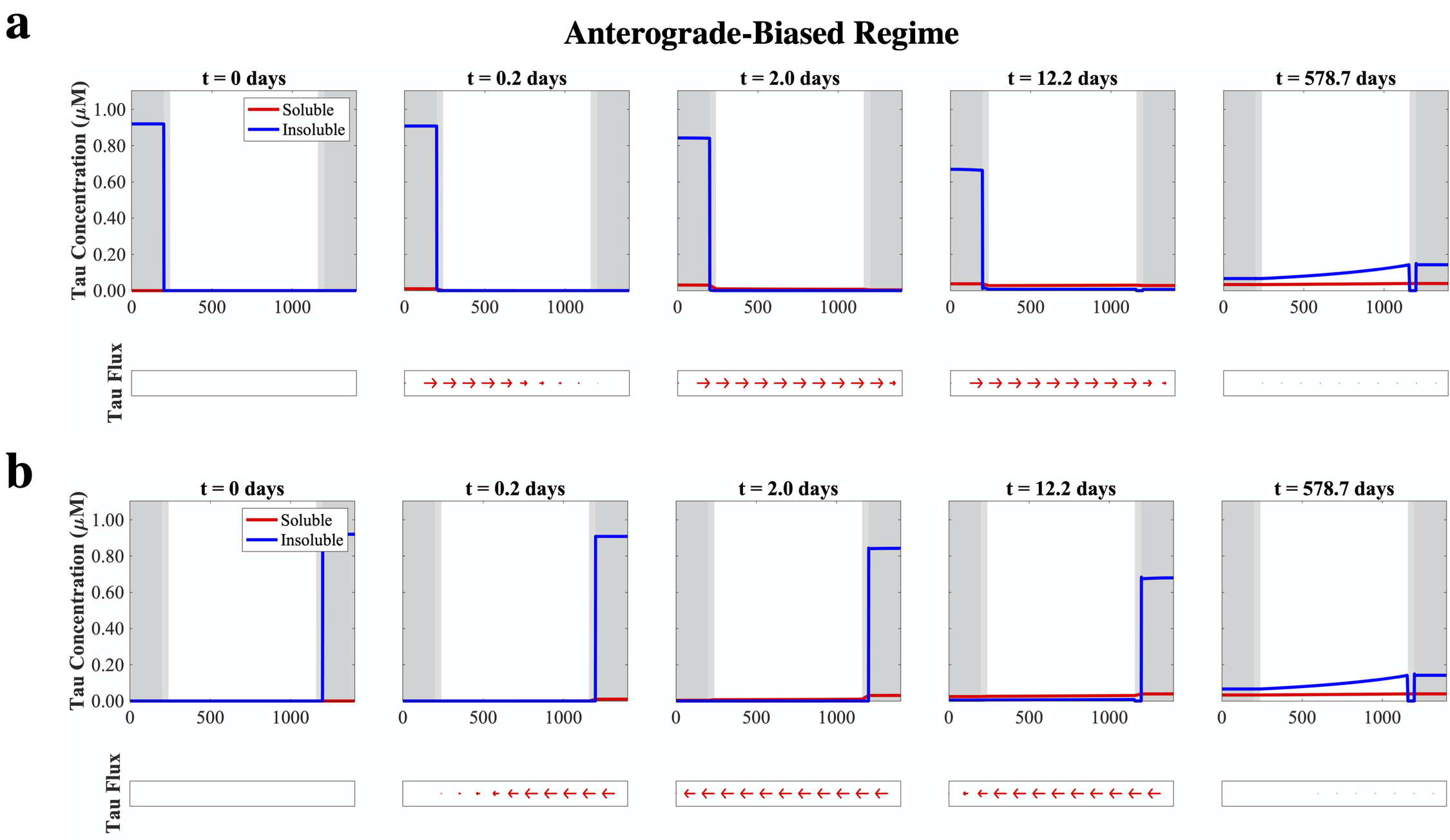

Supplement: S4 Fig — Seeding in either SD compartment converges to the same steady state as the axon-only initial condition in the anterograde-biased parameter regime (Fig 2a). Parameter values used identical to those in S1 Table. (TIF) [file pcbi.1009258.s006.tif]

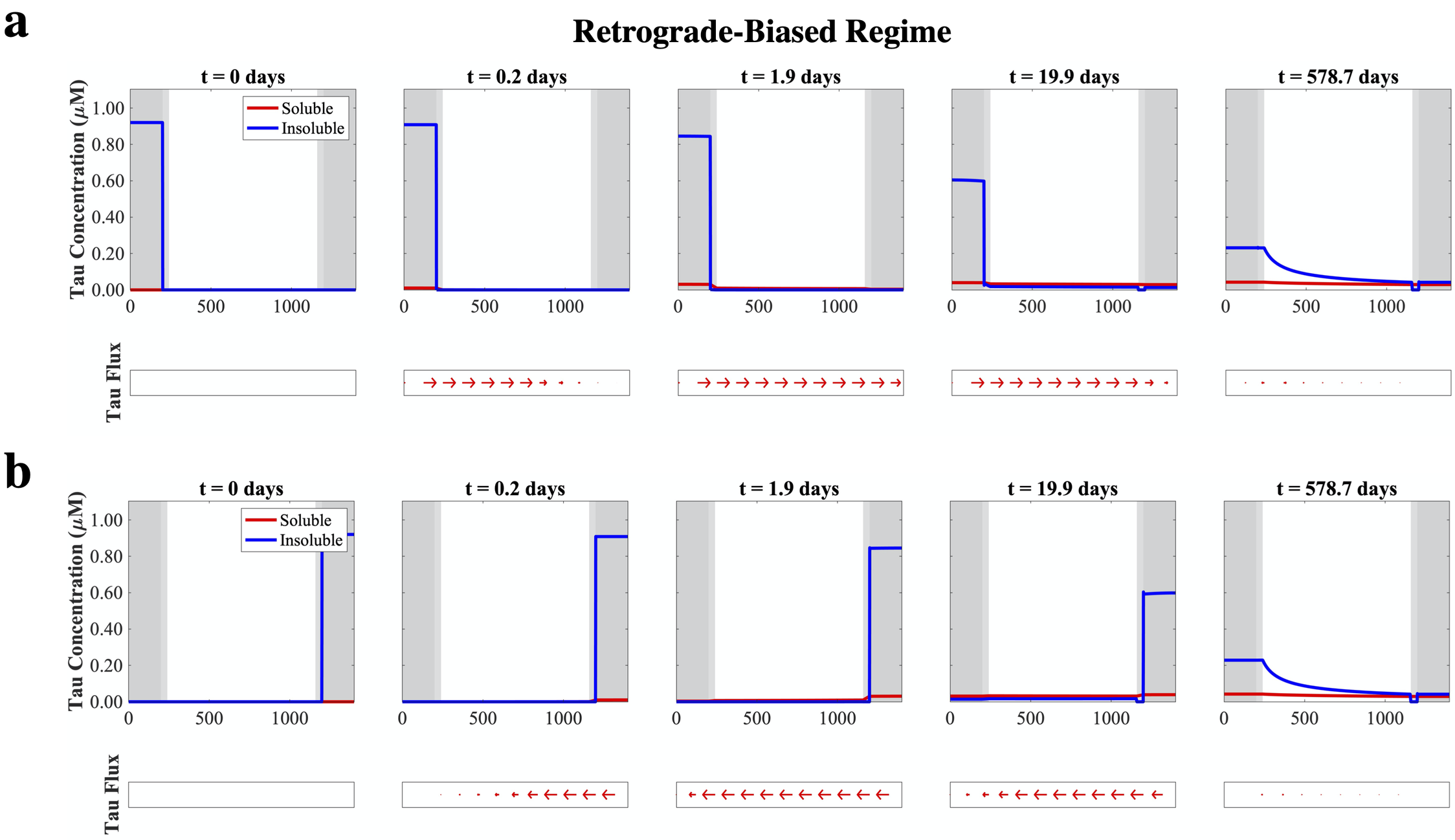

Supplement: S5 Fig — Seeding in either SD compartment converges to the same steady state as the axon-only initial condition in the retrograde-biased parameter regime (Fig 2b). Parameter values used identical to those in S1 Table. (TIF) [file pcbi.1009258.s007.tif]

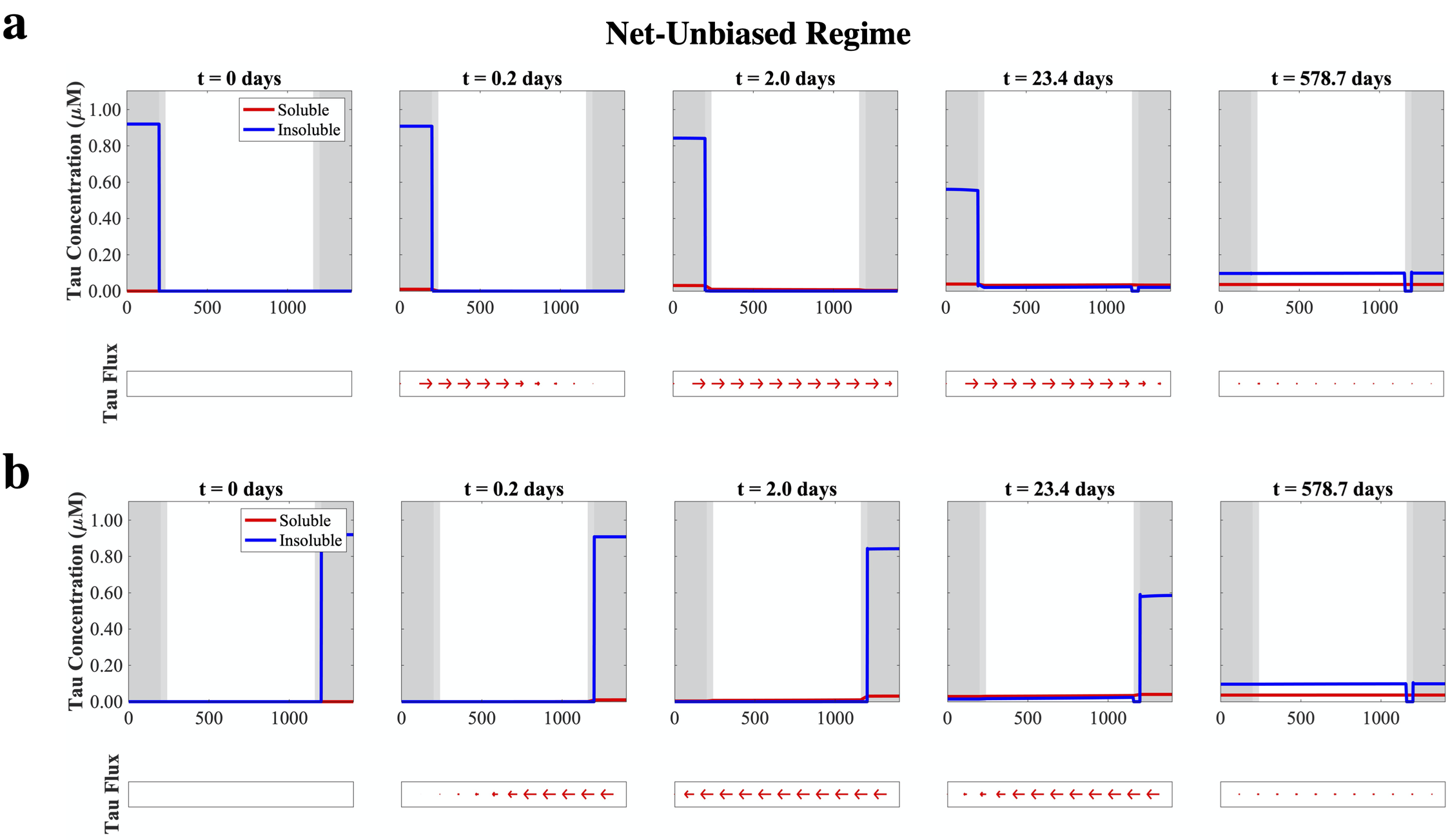

Supplement: S6 Fig — Seeding in either SD compartment converges to the same steady state as the axon-only initial condition in the net-unbiased parameter regime (Fig 2c). Parameter values used identical to those in S1 Table. (TIF) [file pcbi.1009258.s008.tif]

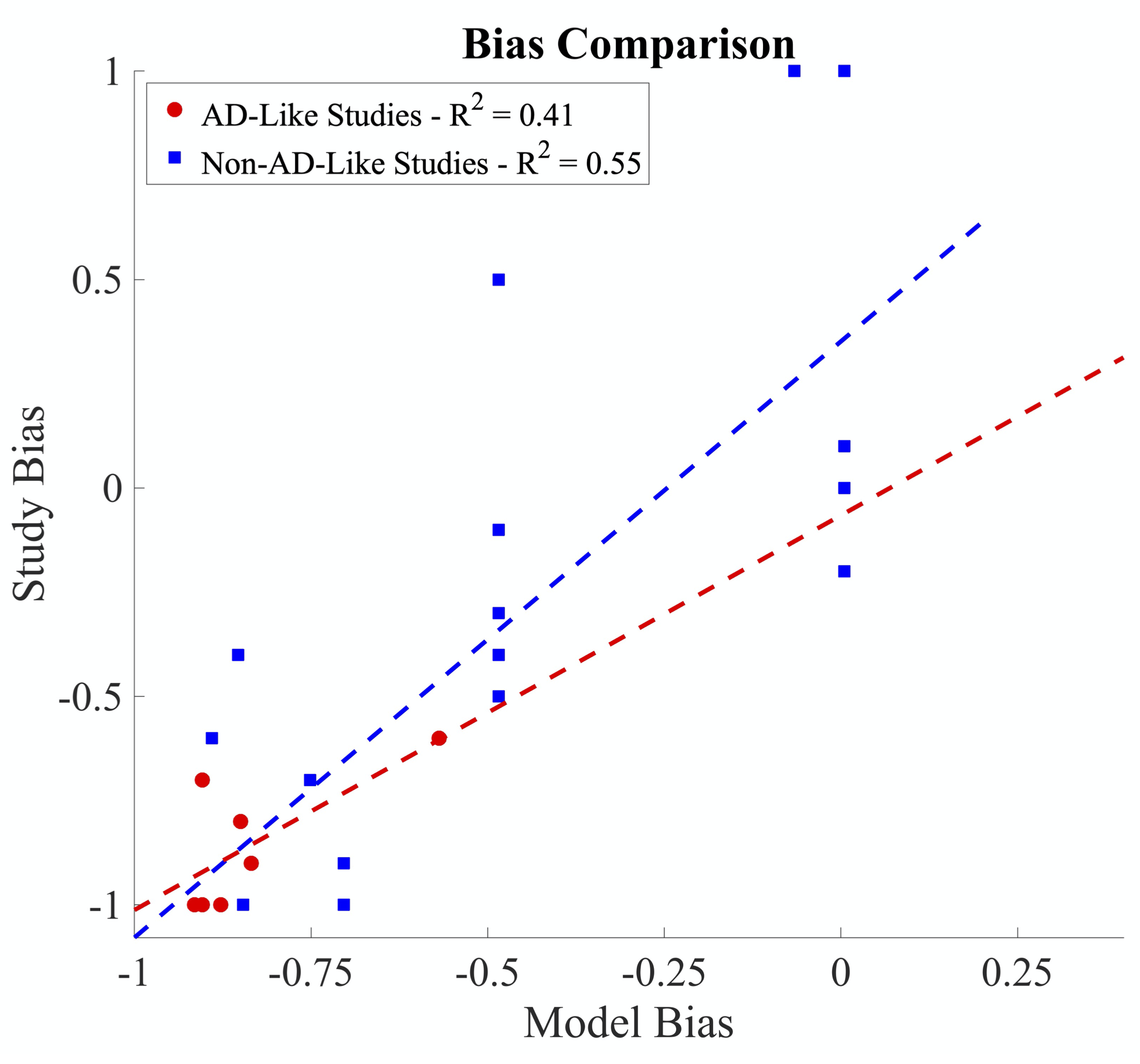

Supplement: S7 Fig — Scatterplot showing the relationship between mouse network bias across several tauopathy studies and the bias predicted by the transport model at equivalent time points. The adjusted R2 values for each set of studies are listed in the legend. (TIF) [file pcbi.1009258.s009.tif]
