# Supplementary material for: Emergence of directional bias in tau deposition from axonal transport dynamics
Source: PLoS Comput Biol. 2021 Jul 27;17(7):e1009258. doi: 10.1371/journal.pcbi.1009258 (PMC8345857; doi:10.1371/journal.pcbi.1009258)
Supplement: Attachment — Submitted filename: PLOS Comp Bio Rebuttal.pdf [file pcbi.1009258.s013.pdf]

June 14th, 2021

To the Editorial Board of PLOS Computational Biology,

Please find enclosed a revised version of our manuscript entitled *"*Emergence of directional bias in tau deposition from axonal transport dynamics*"* by J. Torok, P.D. Maia, P. Verma, C. Mezias, and Ashish Raj. The reviewers raised interesting questions, which led to significant improvement of the manuscript. Therefore, we believe that the review process has greatly strengthened the scope and aim of the paper. In addressing the reviewer's comments, we have alleviated all concerns and feel that the improved manuscript may be ready for publication.

Below is an extensive account of our responses to the reviewer's concerns. An account is also given for the changes made directly to the new manuscript. We hope that such a detailed summary of manuscript changes can aid the process for evaluating the rebuttal process. Our answers to specific questions and comments are shown in blue and modifications/additions to the manuscript are highlighted in red. In the markup version of the revised manuscript, we have also shown changes to the original text in red.

If you desire any additional information, please do not hesitate to contact us at your earliest convenience.

Sincerely,

Justin Torok (jut2008@med.cornell.edu), Pedro D. Maia, Parul Verma, Christopher Mezias, and Ashish Raj.

Original Editorial Response:

Dear Mr. Torok,

Thank you very much for submitting your manuscript "Emergence of directional bias in tau deposition from axonal transport dynamics" for consideration at PLOS Computational Biology.

As with all papers reviewed by the journal, your manuscript was reviewed by members of the editorial board and by several independent reviewers. In light of the reviews (below this email), we would like to invite the resubmission of a significantly-revised version that takes into account the reviewers' comments.

We cannot make any decision about publication until we have seen the revised manuscript and your response to the reviewers' comments. Your revised manuscript is also likely to be sent to reviewers for further evaluation.

When you are ready to resubmit, please upload the following:

[1] A letter containing a detailed list of your responses to the review comments and a description of the changes you have made in the manuscript. Please note while forming your response, if your article is accepted, you may have the opportunity to make the peer review history publicly available. The record will include editor decision letters (with reviews) and your responses to reviewer comments. If eligible, we will contact you to opt in or out.

[2] Two versions of the revised manuscript: one with either highlights or tracked changes denoting where the text has been changed; the other a clean version (uploaded as the manuscript file).

Important additional instructions are given below your reviewer comments.

Please prepare and submit your revised manuscript within 60 days. If you anticipate any delay, please let us know the expected resubmission date by replying to this email. Please note that revised manuscripts received after the 60-day due date may require evaluation and peer review similar to newly submitted manuscripts.

Thank you again for your submission. We hope that our editorial process has been constructive so far, and we welcome your feedback at any time. Please don't hesitate to contact us if you have any questions or comments.

Sincerely,

Michele Migliore, Associate Editor, PLOS Computational Biology.

Kim Blackwell, Deputy Editor, PLOS Computational Biology.

Author Summary of Changes:

In addition to minor typographical changes suggested by the reviewers, we have made the following major changes to the manuscript:

- **Figure 1** has been modified to include a cartoon of the model system.
- **Figure 3** has been modified to include total presynaptic and total postsynaptic tau, so that the plotted bias metric is more intuitive to understand.
- We added **Table 2** summarizing which processes are on/off in each compartment.
- We have **added** a supplemental figure, **Figure S1**, which shows the convergence of each model condition to a single steady state within the time range of the simulations.
- We have added **Methods** Section 4.3, which has multiple subsections detailing various methodological choices that required further explanation. It also defines the bias quantity explored in multiple figures in Equation 15, which was suggested by multiple reviewers as necessary.
- We have changed references to “fixed point” and “equilibrium state” to “steady state,” which was suggested by Reviewer #1 as being more mathematically precise.

All changed text is marked in red font in the markup version of the manuscript. We have also responded point-by-point to each of the reviewer comments below. We appreciate the insightful, detailed feedback from all three reviewers and hope that the changes listed sufficiently address their concerns.

Reviewer #1:

I: Overview:

Torok and colleagues develop a mathematical model of the spatiotemporal evolution of soluble and insoluble tau species to study if changes in axonal transport based on tau species conformer could explain differential behavior observed in tauopathies based on amyloid status.

The mathematical model is of a one-dimensional interval taken to model two neurons, but represents all the critical mechanisms in an elegant way. The results in the manuscript are well explained in words, but the presentation in the Figures was less clear and more confusing than necessary. This work has strong contributions to the field, below I offer comments which if addressed will make its particular results more clear to understand.

II: Major Comments

(1) The critical figure in this paper, Figure 1, could be improved to make it more clear which processes are happening in which compartments. Once I read the model description it made sense, but I think with a few more additions, it could be really clear just by looking at the figure. For example, Figure 1 seems to indicate that diffusion, active transport and interconversion are only happening in the axon.

This is an excellent point. We have added another table to the manuscript [**Table 2**] that summarizes which processes occur in which compartments, which accompanies this figure. We have also included a color-coded cartoon of the system showing the correspondence between the boxes in the schematic [**Figure 1**] to the biological entities they represent, following Comment #3ii:

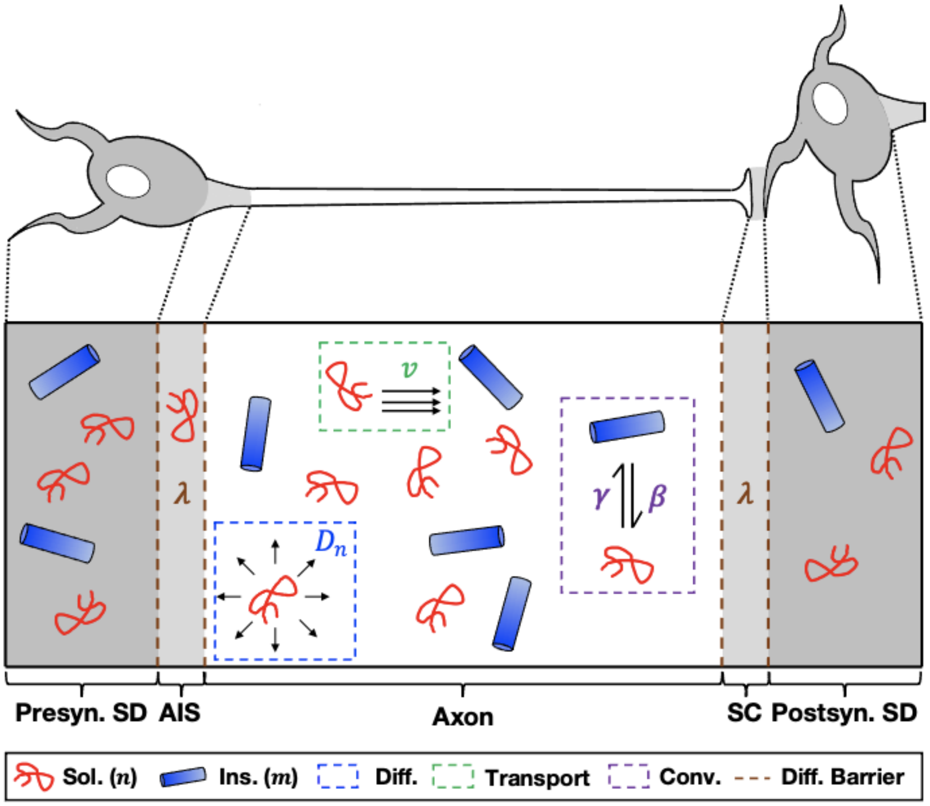

(2) The authors assume conservation of mass in this system. This, of course, makes sense for several reasons (as they write in Section 4.2.2: mathematical simplicity and because they are modeling things on a small time scale). However, in Figure 2 they show results out to 500+ days.

I had difficulty interpreting this late time result in the context of the biology. Is this still a time-scale that we would expect conservation? Was the key purpose in this late time point to simply show that the system reaches a steady-state distribution? If so, I would forgo this to the modeling section or state explicitly.

Yes, the main purpose for simulating the model out to 500+ days was to show steady-state distributions, and it is true that in the *in vivo* context, the mass conservation assumption is violated due to intrinsic recruitment of healthy tau and spread from neighboring neurons, as we state in the **Discussion** (Section 3.3). Mass conservation is assumed here for model

interpretability. We have also added text to **Results** Section 2.2.1 to make this explicit as suggested:

"We depict the concentrations of *n* and *m*, as well as the calculated concentration flux of *n*, j_{net} (obtained by numerically evaluating Eq. 4 post hoc), at five time points between model initialization and the point at which steady-state distributions are established, which typically takes a matter of months in model time with this parameterization (S. Table 1). Although at these long time scales it is unlikely that the assumption of mass conservation made in our model holds, the steady-state behavior of this system is of theoretical and practical importance and explicitly modeling pathological tau recruitment would hinder the model's interpretability."

In addition, I looked for a practical definition of the Tau Flux quantity that was at the bottom of the Figure. (See point 4 below)

Tau flux, j_{net}, is numerically evaluated using Eq. 4 after obtaining the simulation results from pdepe, which is distinct from bias. The **Results** text referred to j_n which was confusing - we have changed this to j_{net} and made a reference to Eq. 4. Note that tau flux is distinct from bias, which we should have defined explicitly in the manuscript and have now done so in Eq. 15.

(3) I had a number of problems interpreting Figure 3:

(i) The bias (magenta line) in Figure 3 is confusing. This is partly because it's not adequately defined in the caption. But I remained confused by its definition in the text of the manuscript.

Part of this is because I'd wanted to see an equation in terms of the model variables (and of course the model occurs in a later section).

We have added Eq. 15 to the **Methods** to define this quantity explicitly.

Even though it would make the figure more complicated, I would have preferred to see (rather than the bias) which is somewhat difficult to interpret, the total presynaptic tau (soluble + insoluble) and postsynaptic tau (soluble + insoluble) plotted as well.

The bias then would be directly visible and clear (rather than rely on this more indirect assessment)

The reason for plotting bias is to introduce it as a quantity of interest to be tracked in further Figures (namely **Figures 4 & 6**). While we feel it is important to show this quantity, we have broken up the original **Figure 3a** into two sets of panels as shown below, adding the total somatodendritic tau in the presynaptic and postsynpatic neurons as suggested along with the bias to make its meaning more visually intuitive.

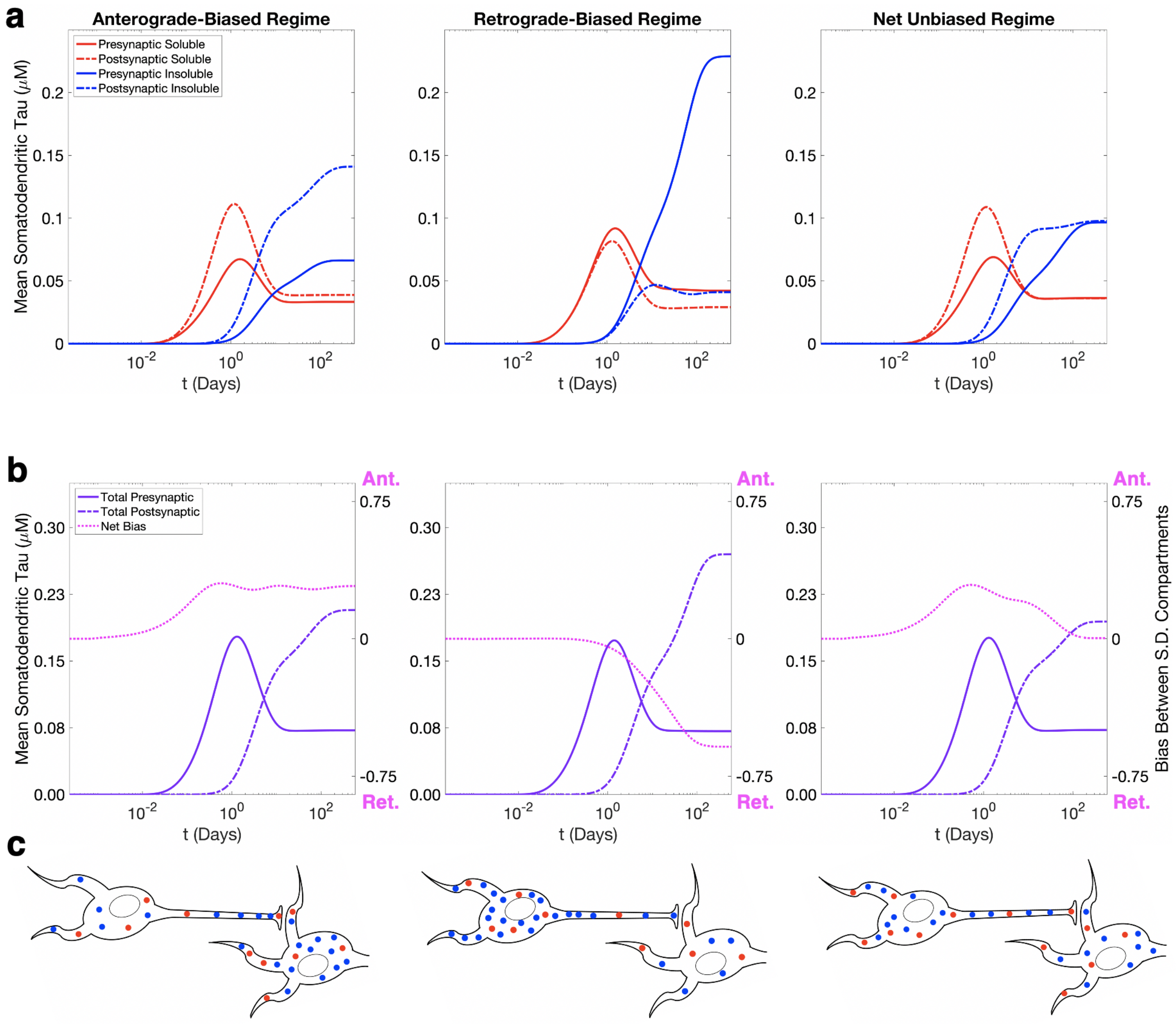

(ii) The figure shown in panel (b), while informative, is confusing in the present context because this is the first presentation of neurons in this manuscript!

Since not all readers of PloS Computational Biology will be familiar with neurons (and what is meant by the synapse) it would actually have been helpful to include a schematic version of a neuron in Figure 1. Or at the very minimum - label the regions in Figure 3.

We have made this change to **Figure 1** so that introducing the schematic here is more understandable.

(4) Figure 4 too was challenging to understand precisely. Overall the behavior shown in the plots made sense as did the authors' interpretation of it.

(i) I could not determine the precise quantity that was plotted. I suspect that this is in fact the bias quantity in Figure 3 and perhaps related to Tau Flux in Figure 2. Again, a specific equation for SD bias would have been helpful.

As mentioned above, we have added Equation 15 to the **Methods** to define this quantity explicitly. It is not an explicit function of the soluble tau flux in **Figure 2**, which is defined in Eq. 4.

(ii) Finally, since the authors do not have explicit steady-state solutions to this model, I presume this bias quantity was calculated numerically at some future time?

We take the bias evaluated at the last time point, where tau distributions are stable if not explicit steady-state solutions to the model equations. However, this raises an important point (further enumerated upon in Comment #5) about the assessment of the extent to which the model has settled into a steady state. We have added a calculation of the relative rate of change of the model distributions near the final time point to demonstrate that they are appreciably stable; see **Methods** Section 4.3.1 and new **S. Figure 1**.

(iii) I presume that these lines were numerically fit? If so, at least a sentence about how would be helpful.

We use a two-step process to compute the lines of zero-bias from the 2D array of steady-state bias values (each corresponding to a different (δ, ϵ) feedback parameter pair), represented by the heatmaps in **Figure 4**. First, at every δ value in this array, we fit a cubic polynomial to the vector of bias values and find its real root in the [0, 1] range of ϵ. These (δ^*, ϵ^*) pairs define a manifold in parameter space where bias is zero. We then fit a linear polynomial to these points to obtain the equations displayed in **Figure 4**. We have added text to the **Methods** (Section 4.3.3) enumerating this procedure.

(5) While the present scope of the study sought a numerical analysis of this model, as a mathematician I am intrigued by the possibility of analytical results for this model. The model presented is globally attractive, and it seems that this steady-state distribution might be possible to determine analytically at least for some regimes/conditions.

This is a fantastic comment! Indeed, by setting u(x,t) → U(x) (where u(x,t) = n(x,t) or m(x,t)) as t→∞, one arises on a boundary value problem (BVP) for this steady-state profile. Since this system of equations involves the second (spatial) derivative, we can write y1 = N(x), y2 = dN/dx , y3 = M(x), and y4 = dM/dx, which yields y' = F(y) along with the corresponding BCs. This formulation follows the syntax required by MATLAB's bvp4c solver, which we tentatively implemented. However, we encountered some challenges that preclude us from expanding this further in a timely manner for this work, such as:

- Using the profile obtained at the last time simulated by the PDE solver (pdepe) as initial guess for bvp4c is challenging due to the sharpness/discontinuities of the profile in the

spatial dimension. In particular, the profile is extremely sharp at the synaptic cleft, where the aggregation of soluble tau to insoluble tau is forbidden.

- The PDE solver appears to handle sharpness in the profiles much better than bvp4c.
- The RHS equations for y2' and y4' are very convoluted and prone to numerical inaccuracies.

We would be very interested in future analytical investigations regarding this topic, particularly if one cast the problem as a singularly perturbed bvp or boundary-layer theory.

For now, in order to (numerically) justify the steady-state distribution nomenclature used in this work, we provide the following additional plots in the SI and refer to them accordingly throughout the text.

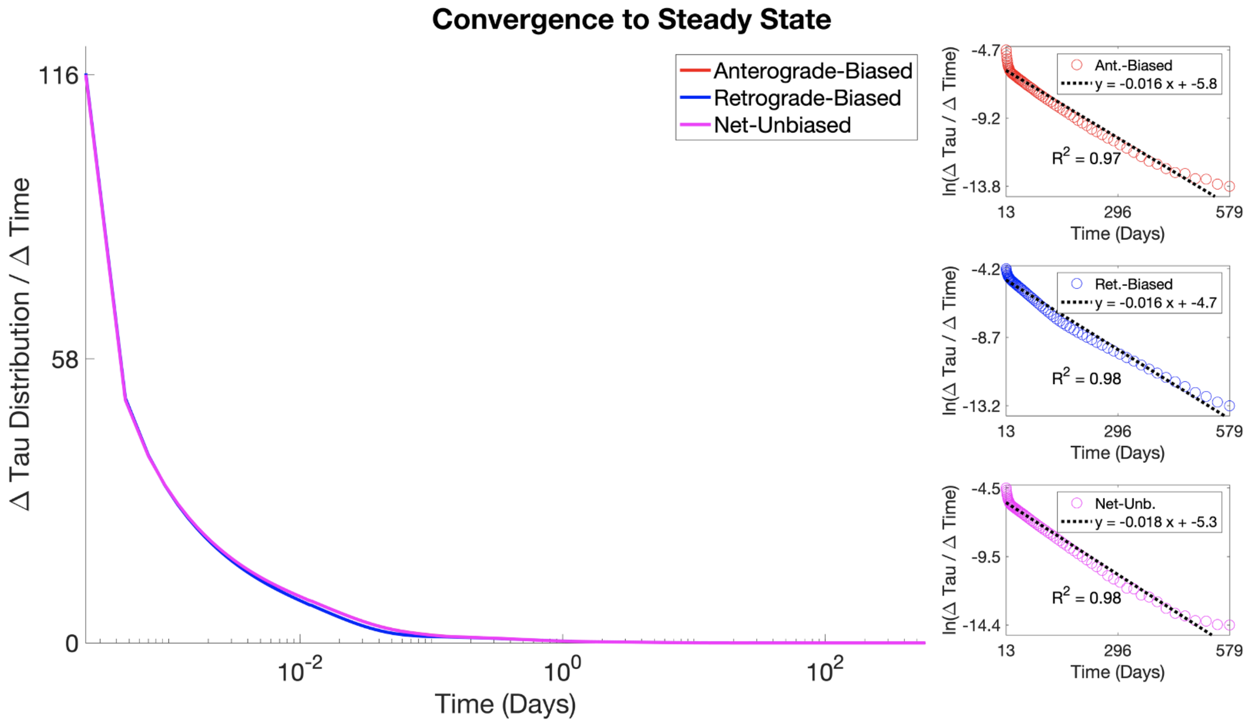

S. Figure 1 shows the relative changes in tau spatial profile over time for different parameter regimes. We numerically estimate the rate of change of the tau concentrations at consecutive time steps (see **Methods** Section 4.3.1) and plot this quantity versus time in days (left panel). The plots demonstrate that the spatial profiles will change very little after sufficiently long times, and that at the final evaluated time point the distributions are essentially unchanging. Interestingly, at mid-to-late model times, the convergence to steady state is approximately exponential, with quantitatively similar rates of decay (right panels).

Finally, the authors (Figure 5) refer to a "fixed-point". That is more commonly used for ODEs. Since the authors are using a PDE, the term "steady-state distribution" or "steady-state density" would be more appropriate.

We have changed the text accordingly.

III: Minor Comments & Typos

We have addressed all of the following minor issues below as recommended by the reviewer.

(1) In Table 1, I'd like to have a little more clarity of where the estimated parameters came from. The authors cite [34], but something such as, "Estimated by fitting a model to mouse data", "Estimated from in vitro assays". Something to give your readers the understanding of where the values came from.

(2) Page 3/4: Change "biological compartment it resides" to "biological compartment in which it resides"

(3) Page 7: Change "by total somatodentric" to "by total somatodendritic

(4) Page 9: Change "with these biological plausible conditions" to "with these biologically plausible conditions"

(5) Page 14 in the Model section has a number of Typos:

(i) Change: "demonstrate how the it influences model" to "demonstrate how it influences the model"

(ii) I recommend replacing the following text: "by modeling these compartments with a 200 m one-dimensional lengths."

With this: "by modeling these compartments as a one-dimensional segment 200 microns in length."

(iii) Aren't there two SD compartments?

Change: "The dynamics of the SD compartment only differs"

To: "The dynamics of the SD compartments only differ"

(6) Page 15:

Change: "that allows tau migrate" To: "that allows tau to migrate"

Reviewer #2: In their manuscript, entitled “Emergence of directional bias in tau deposition from axonal transport dynamics" by Torok et al., the authors propose a first model to explain the alterations in axonal transport due to pathological Tau forms based on the differential impact of soluble and aggregated species. The model developed foresees the profile of tau distribution between a pair of pre- and postsynaptic neurons, and seems to be robust and stable through different initial conditions. Its elegance lies on the fact that the ratio between a few of its parameters easily describes the biases recovered from the simulations. The authors present also an initial correlation to in vivo studies, and they also recognized the precariousness of these comparisons based on the model´s limitations.

Altogether, the present work offers a good first model for understanding the initial states of AD and Non-AD related pathologies where the first affected neuron (with the axon compartment) passes on the pathological tau forms to the next one (or eventually previous one). This could serve as a starting point for designing future studies in order to gain molecular insight for tauopathies. The authors describe several restrictions of the model, such as a constant concentration of pathological tau species (n + m) over a period of days-months-years. Applicability of the models may increase if these corrections are introduced, as well as with the introduction of alternative boundary conditions. It would be of particular interest to analyze if the profiles are also reproduced at a larger scale, for example, introducing periodic or non-homogeneous conditions to simulate a larger “wire” of neurons with a mismatch between entry and exit of tau species at the SD compartments. However, the complexity of these models may require a dedicated manuscript.

A few minor concerns are described below:

- Section 2.2.2. Reconsider position of second parenthesis in the phrase: “…opposite direction (epsilon >> delta; Fig. 2b) and Vid. S2.”

Fixed!

- Labeling of supplementary videos SVideo_nob_regime.mp4 and SVideo_ret_regime.mp4 is inverted.

Fixed!

- Section 2.2.3. Second parenthesis is missing in the phrase “…both delta and epsilon values to be significantly greater than 0 (Fig. 2c and Vid.”

Fixed!

- Section 3.3. Referring to a plasma membrane as simple phospholipid bilayers is usually a big oversimplification. Considering the phrase “…migration across the two phospholipid bilayers of the SC…”, pre- and post-synaptic membranes are particularly of high complexity and its content

of proteins is above average and not easy to overlook, particularly when referring to more complex mechanisms of transport for pathological Tau forms.

We have reworded this point so as to not downplay the complexity of the synapse:

“It is likewise inaccurate to assume that it is a purely diffusive process that facilitates tau migration across the cell membranes on either side of the SC…”

- Section 4.1.1. The expression “… and therefore demonstrate how the it influences model behavior…”, should be changed for “… and therefore demonstrate how it influences model behavior…”.

Fixed!

Reviewer #3: The authors are interested in investigating how different tau conformers may lead impact active transport and lead to different neurodegenerative pattern. They develop a 1-dimensional PDE model of tau protein transport in different axonal and synaptic compartments. Specifically, they simulate soluble and insoluble tau spreading between two neurons. The model has several strong simplifying assumptions that could be better justified. Model parameters (such as tau transport feedback, aggregation, fragmentation parameters) are varied to study the balance between anterograde and retrograde bias in the tau protein distribution. The authors apply this model to Alzheimer disease and non-AD in vivo mouse tauopathy models; the comparison is really to a network model from the same group, which should be made clearer. I appreciate that the authors provide a well-documented Github repository for their computational model.

Suggested revisions:

1. In most modeling studies for intracellular transport, effective transport is a quantity that emerges from the interplay of dynamics between anterograde and retrograde transport, diffusion, and pausing behavior. Here, it is used to mean an expression for velocity in the active transport state only as a function of two parameters introduced in this study. I would suggest reconsidering or clarifying the terminology to better connect to other studies.

We agree with the reviewer. To accommodate this important suggestion, we have edited Section 2.1 of the **Results** in the following way:

"These two tau species can interconvert through the opposing processes of fragmentation and aggregation (Eq. 5). For both biophysical and mathematical (see for instance the work of Kuznetsov and Kuznetsov [35]) reasons, we model the effective transport of the mobile, soluble species as two component processes: diffusion and active transport, the latter of which only occurs within the axonal compartment. The effective velocity of active transport within the axon, v (Eq. 1), is a function of the local concentrations of the soluble and insoluble species, because each can perturb the effective anterograde transport rate of kinesin [28,29,31]. Specifically, we allow soluble tau to increase effective anterograde transport velocity through the parameter δ and insoluble tau to decrease it through the parameter ϵ (Table 1). Since the effective retrograde transport velocity is unaffected by pathological tau [26,27], directional bias of tau transport is governed by the balance of δ and ϵ acting upon anterograde transport rate. Diffusion between the SD and axonal compartments is hindered by the physical barriers of the AIS and SC, leading to slow, concentration-gradient-dependent mixing."

We have also edited the beginning of Section 3.1 of the **Methods** to mention how the modeled processes relate to effective transport:

"We use a coupled system of partial differential equations (PDEs) to model the concentrations of two species of pathological tau: soluble n(x,t), which can travel via *diffusion* and *active transport* processes (the net balance of which determines the effective transport of pathological tau), and insoluble tau m(x,t), which is immobile."

2. As the authors mention in 4.1, the dynamics of motor proteins follows a start-and-stop dynamics; such a model has been validated with experimental data for axonal transport of various protein cargoes. It would be useful to mention in the paper how this simplifying assumption could affect the insights from this model.

Although the start-and-stop dynamics of the axonal motor proteins are instrumental to understanding their functioning on short time scales, at the longer time scales (on the order of days) we explore here, we posit that the net movement of cargoes by kinesin and dynein can be modeled with effective average velocities in the anterograde and retrograde directions, respectively. We anticipate that explicitly modeling of motor behavior, in addition to greatly adding to the computational complexity, will have little to no effect on the steady-state distributions obtained with the effective velocity model we use in the manuscript. This is an important point and we have added the following text to Section 4.1 to elaborate on it:

"In this expression we have assumed that on the time scale of the simulation (days), the characteristic "start-and-stop" dynamics of molecular motors, where motor binding and unbinding events happen within fractions of a second [53], can be subsumed into effective velocity parameters. We anticipate that the separation of time scales between characteristically "slow" processes such as aggregation and fragmentation and the "fast" dynamics of motor-microtubule interactions allows us to time-average the latter without a loss of generality."

3. I understand the need to keep the model 1-dimensional for computational efficiency. I recommend rephrasing some parts of section 4.1.2, such as "While the dynamics within axons can reasonably be collapsed into one dimension, those within the SD compartments cannot." Since the authors make such a simplification to one dimension in this study (given the quantities of interest to them), they should instead start by explaining why their goals allow them to make this approximation.

This is an important point and we have rephrased Section 4.1.2 accordingly:

"Our two-neuron system incorporates two somatodendritic (SD) compartments, corresponding to the presynaptic and postsynaptic cell bodies, which are modeled identically. For the purposes of this model, we focus on the overall amounts of SD tau - particularly the differences between presynaptic and postsynaptic neurons - that result from the tau dynamics within the axon. Although a one-dimensional model cannot be used to precisely model the spatial distributions of tau in these compartments, which have complex, three-dimensional geometries, a one-dimensional approximation is sufficient to capture the mean somatodendritic tau concentrations. We therefore greatly simplify the system by modeling these compartments as one-dimensional segments 200μm in length. The dynamics of the SD compartments only differ from the axonal compartment regarding the active transport of n. We do not expect transport to be more efficient than diffusion in the soma, and transport in the dendrites is both distinct from that in the axons and beyond the scope of our model."

4. In section 2.3, it would be useful to provide the bias measure in equation form. I am also unsure if figure 3b) best captures the insights from this section, but perhaps this is simply my stylistic preference.

It was an omission to not have written the bias in equation form, and we have added **Equation 15** to the **Methods** to define this quantity. We have also modified **Figure 3** to make it less condensed as another reviewer suggested, moving the original panel (b) to (c) and adding plots of the total somatodendritic tau in each compartment, which is directly used to calculate bias.

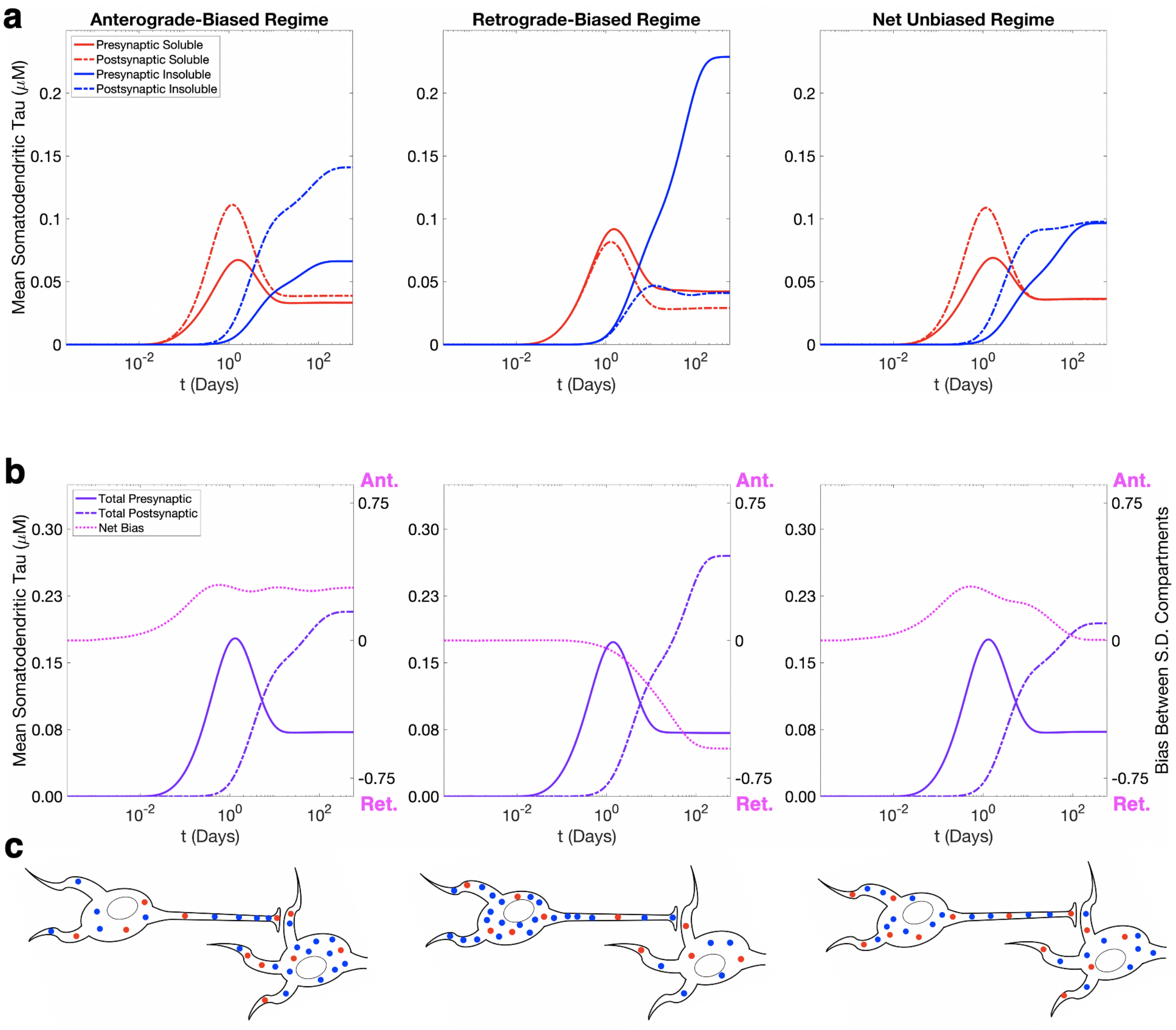

5. For section 2.4: it is not surprising that a combination of the transport parameters influences the final state of the system, given the expression in equation (1).

It is indeed the anticipated behavior of the model that the steady-state bias between somatodendritic compartments is governed by an interplay of the feedback parameters. However, the linear relationship between δ and ϵ demonstrated in **Figure 4**, as well as the further dependence on the aggregation and fragmentation parameters, is not easily gleaned from a cursory inspection of the model equations. The main point in Section 2.4 is that, at steady state, bias can be expressed as a simple linear function of the ratios of these parameters.

6. In Table S1, it would help to add the literature sources for the model parameters used.

We have added a reference for the parameters in this table that were derived from the literature. The majority of these parameters, however, lack experimental evidence that is directly applicable to the system in question and they were chosen heuristically to demonstrate the dynamics of the system. We have added Section 4.2.3 to the **Methods** to clarify this point, which states the following:

"For the effective transport parameters that were estimated in a comparable *in vitro* system, we rely upon the experimental work of Konzack, *et al.* in cultured rodent neurons [35]. The interconversion parameters in Table S1, which are more challenging to estimate with high precision in a cellular context, were tuned to yield biologically plausible rates of insoluble tau accumulation. The transport feedback parameters, whose impact on model behavior is the primary focus of the present work, were each varied within a range of 0 to 1."

7. The authors repeatedly refer to "biases at the network level" in this manuscript. It seems that this is related to another preprint describing a network-level model from their group, but it is unclear what this phrasing means without reading that study. I think it is important that they clarify this and that they mention that the results are compared with results another modeling study (applied to experimental datasets). Since they compare directional biases across these models in Figure 6, I recommend that they also summarize how bias is quantified in that model.

We have added the following sentence to the **Results** section describing how these network bias metrics were obtained by Mezias, *et al*. - we agree that we should make that clearer:

"In this study, network bias was parameterized as the extent to which the spread of tau over the brain network is biased along retrograde-directed or anterograde-directed connections and its value was fit at each experimental time point using regional tau deposition data from a wide variety of mouse tauopathy models."

8. In section 2.6, how were the parameters chosen in the simulations that match the directional bias data from in vivo studies? Did the authors carry out parameter estimation, or vary their parameters over some ranges? More details should be given here, especially since multiple parameter combinations might give good fits to this data.

We varied the parameters over reasonable ranges relative to the values used in **Table 1** to achieve these fits and we have added Section 4.3.5 to the **Methods** elaborating on our methodological choice here:

"We varied β, γ, δ, ϵ, and *f* within reasonable ranges ([1×10^{-8}, 1×10^{-5}] for β and γ; [0,1] for δ and ϵ; and [0.5,1] for *f*) to find parameterizations with good fits to network bias data so that we could demonstrate that the model can plausibly recreate the time dependence of directional bias. The parameterizations in S. Table 2 do not uniquely identify the models of best fit; however, the differences between the two sets of parameters remain (e.g. higher γ to β ratio for AD-like mouse models) consistent for other parameterizations with comparable fits. We chose these

specific parameterizations because they achieve excellent concordance with network bias and differ only in three parameters (β, γ, and δ)."

We also add the following text to the end of the **Results** Section 2.6:

"Given the relative paucity of data against which we compare the transport model, we cannot claim that these parameterizations uniquely fit each set of tauopathy models. Rather, our model highlights two biophysical mechanisms - aggregation to fragmentation rate ratio and differential interactions between soluble tau and kinesin - that can explain the differences between the network biases that develop in AD-like and non-AD-like mouse models."

The reviewer is correct to point out that there are multiple parameter combinations that achieve similar fits to data; however, as stated above, the points made in the text about the differences in parameterization between AD-like and non-AD-like mouse models still remain valid. We have chosen these two particular parameterizations because they are parsimonious in terms of the number of parameters that differ between them.
